# Exploiting the Autofluorescent Properties of Photosynthetic Pigments for Analysis of Pigmentation and Morphology in Live *Fremyella diplosiphon* Cells

**DOI:** 10.3390/s100706969

**Published:** 2010-07-19

**Authors:** Juliana R. Bordowitz, Beronda L. Montgomery

**Affiliations:** 1 Plant Research Laboratory, Department of Energy, Michigan State University, 106 Plant Biology Building, East Lansing, MI 48824-1312, USA; E-Mail: jbordowitz@ucsd.edu; 2 Cell and Molecular Biology Program, Michigan State University, 2240A Biomedical and Physical Science Building, East Lansing, MI 48824-4320, USA; 3 Division of Biological Sciences, University of California-San Diego, La Jolla, CA 92093-0116, USA; 4 Department of Biochemistry and Molecular Biology, Michigan State University, 210 Biochemistry, East Lansing, MI 48824-1319, USA

**Keywords:** autofluorescence, confocal laser scanning microscopy, cyanobacteria, fluorescence imaging, light, microscopy, morphology, phycobiliproteins

## Abstract

*Fremyella diplosiphon* is a freshwater, filamentous cyanobacterium that exhibits light-dependent regulation of photosynthetic pigment accumulation and cellular and filament morphologies in a well-known process known as complementary chromatic adaptation (CCA). One of the techniques used to investigate the molecular bases of distinct aspects of CCA is confocal laser scanning microscopy (CLSM). CLSM capitalizes on the autofluorescent properties of cyanobacterial phycobiliproteins and chlorophyll *a*. We employed CLSM to perform spectral scanning analyses of *F. diplosiphon* strains grown under distinct light conditions. We report optimized utilization of CLSM to elucidate the molecular basis of the photoregulation of pigment accumulation and morphological responses in *F. diplosiphon*.

## 1. Introduction

Photosynthetic organisms depend upon light for carbon fixation and production of reductant. Thus, the ability to adapt to changes in the photoenvironment is critical. These organisms have diverse mechanisms for light perception and exhibit a number of metabolic and developmental photoresponses. To understand the dynamic molecular processes used for adapting to changes in ambient light, we are investigating the functions of biliproteins, light-absorbing pigments centrally involved in both photosynthesis and the regulation of photomorphogenesis in cyanobacteria, algae, and plants. Photomorphogenesis is the control of growth and development by light intensity and color. The freshwater filamentous cyanobacterium *Fremyella diplosiphon* exhibits a well-known light-dependent acclimation process known as complementary chromatic adaptation (CCA). During CCA, *F. diplosiphon* exhibits light-dependent changes in the protein composition of the photosynthetic light-harvesting complexes, *i.e.*, phycobilisomes (PBSs), and cell and filament morphologies [1]. These changes occur maximally in response to green and red light. Phytochrome-related biliprotein RcaE is responsible for regulating the observed light-dependent changes in PBS protein composition and morphology in *F. diplosiphon* [2–4].

The changes in the PBSs that occur during CCA consist of changes in the phycobiliprotein (PBP) content of the PBSs. *F. diplosiphon* PBSs consist of a core that contains allophycocyanin (AP; wavelength of absorbance maximum [λmax] ∼650 nm) and rods that consist of red-light (RL)-absorbing constitutive phycocyanin (PCc; λmax ∼620 nm) in the core-proximal portion of the rods and inducible PC (PCi) or green-light (GL)-absorbing phycoerythrin (PE; λmax ∼560 nm) in the outermost portion of the rods (reviewed by [5]). The PBSs contains PCi when grown in RL and PE under GL. The PBSs are attached to the thylakoid membrane and transfer energy to chlorophyll *a* (Chl *a*) in the reaction centers of photosynthetic photosystems (reviewed by [5]). By nature of their spectral properties, PBPs are also highly autofluorescent proteins. The three PBPs found in *F. diplosiphon* PBSs exhibit distinct wavelengths of fluorescence maximum (λem): AP, λem ∼660 nm; PC, λem ∼625–645 nm; and PE, λem ∼575 nm (reviewed by [6]). The Chl *a* pigment also is autofluorescent and has λem ∼630–720 nm, which overlaps with PC and AP (see [7] for representative scans).

Microscopy has served as a vital tool for providing insight into cellular structure and function. Many types of microscopy have been used with cyanobacterial systems to provide insight into cyanobacterial characterization and organismal and community structure [8]. General light microscopy has been used widely for gross analyses of cyanobacteria and species classification. Although scanning electron microscopy (SEM) has been used extensively for investigating surface structures of cyanobacteria, and transmission electron microscopy (TEM) has been utilized to explore internal cellular structures [8], confocal laser scanning microscopy (CLSM) has gained popularity for a number of its positive attributes. CLSM is particularly useful as it does not require the chemical fixation or cryofixation methods that are central to the use of SEM and TEM, and thus allows for non-destructive, or live, imaging of individual cells or populations of cells [8]. CLSM also allows imaging of fluorescent, or light-emitting, compounds. Fluorescent proteins have been used in many systems to study cellular development and differentiation, including eukaryotic systems from animals to plants (reviewed by [9]), as well as prokaryotic systems (reviewed by [10]). CLSM technology has proven particularly useful for imaging of cyanobacteria, as these organisms possess highly abundant autofluorescent PBPs and Chl *a*, which can be used as intrinsic fluorescent markers [2,7,8,11–13]. However, heterologous fluorescent probes can also be used with these organisms, despite the presence of intrinsic autofluorescent proteins (for examples see [14–17]). Furthermore, the PBPs have been widely used in heterologous systems as fluorescent protein tags (reviewed by [9]).

Notably, TEM and SEM have been used to study the structure of isolated PBSs from *F. diplosiphon* [18]. More recently, we have adapted the use of CLSM for studying morphology and PBP content of *F. diplosiphon* during CCA *in vivo* [2,11,19,20]. Here, we report on the optimization of the use of CLSM in *F. diplosiphon* for the analyses of pigmentation and cellular morphology using the intrinsic autofluorescent properties of PBPs. We investigated differential laser excitation and collection of emission data to optimize PBP detection and localization, as well as cellular and filament morphology analysis. These studies resulted in the identification of conditions that allow detailed investigations into the molecular bases of the regulation of distinct aspects of CCA through comparative CLSM analyses of wild-type and mutant strains of *F. diplosiphon*.

## 2. Experimental Section

### 2.1. Strains and culture growth conditions

The SF33 strain, a shortened filament strain of *Fremyella diplosiphon* [21], was used as wild-type (WT) pigmentation strain, and the Δ*rcaE* mutant, which is deficient in the photoreceptor that regulates CCA responses, was previously described [2–4]. Strains were grown at 28 °C in BG11 medium supplemented with 20 mM HEPES (BG11/HEPES) shaking at 175 rpm, as described previously [2]. Broad-band green light (GL) and red light (RL) sources were those previously described [2]. A Li-Cor light meter (model LI-250, Li-Cor, Lincoln, NE) connected to a Li-Cor quantum sensor (model LI-190SA) was used to measure light intensity.

### 2.2. Slide preparation

**Figure 1.**
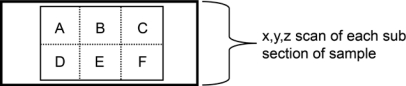
Slide preparation and imaging scheme. Population Z-series scans are obtained by scanning immobilized *Fremyella diplosiphon* cells in the x, y, and z axes for each sub-section of the cover-slip covered area (e.g., A, B, C, D, E, and F) on a slide.

Slides of live, immobilized *F. diplosiphon* cells were prepared using an adapted procedure from Reize and Melkonian [22]. A stock solution of 2% (w/v) UltraPure™ Low Melting Point (LMP) agarose (Invitrogen) was prepared in BG11/HEPES culture medium. The mixture was dissolved fully by heating and mixing on a heated stir plate to homogeneity. LMP agarose cooled to 37 °C in a shaking incubator was added to cells at an optical density at 750 nm (OD_750_) of ∼0.2 to achieve a final OD_750_ of ∼0.1, which yields a final LMP agarose concentration of 1.0%. A 50-μL aliquot of the suspension was pipetted onto a 1.0-mm thick 3″× 1½″ Propper Bev-L-Edge^®^ pre-cleaned twin-frost^®^ slide into a vacuum lubricant-enclosed square (Propper, Long Island City, NY). A cover slip was placed over the suspension (Figure 1). Slides were fixed at 4 °C for 10 mins prior to imaging.

### 2.3. Confocal imaging parameters

Immobilized *F. diplosiphon* cells were imaged with an inverted Axiovert 200 Zeiss LSM 510 Meta confocal laser scanning microscope (CLSM; Carl Zeiss MicroImaging, Thornwood, NY) using Nomarski or differential interference contrast (DIC) optics with a 20× dry objective and fluorescence excitation/emission filters with a 40×/1.3NA oil immersion Plan-Neofluar objective lens or 63×/1.4NA oil immersion Plan-Apo objective lens, as indicated. DIC imaging was conducted with excitation by a 488-nm argon laser at 10% power, using an NT 80/20 filter as described [2]. Initial autofluorescence settings for chlorophyll detection were based on settings adapted from previously published methods [13]. To identify conditions designed to optimize detection of PBP autofluorescence under distinct light conditions, spectral scanning of WT RL- and GL-grown cells was initiated.

Optimization of PBP and Chl *a* autofluorescence detection for *F. diplosiphon* was conducted using 488-, 543-, and 633-nm lasers for excitation. For 488-nm excitation, the argon laser was set at 10% power and the images were collected with an NT 80/20 filter. Emission scans were collected in ∼10.7-nm bandwidth increments in the range from 500 nm to 750 nm. For 543-nm excitation, the helium-neon laser was set at 30% power and the images collected with an NT 80/20 filter. Emission scans were collected in ∼10.7-nm bandwidth increments in the range from 550 nm to 750 nm. For 633-nm excitation, the helium-neon laser was set at 10% power and the images collected with an NT 80/20 filter. Emission scans were collected in ∼10.7-nm bandwidth increments in the range from 640 nm to 750 nm. Acquired CLSM images were obtained with the LSM FCS Zeiss 510 Meta AIM imaging software.

## 3. Results and Discussion

### 3.1. Determining imaging parameters for *F. diplosiphon* PBP autofluorescence

**Figure 2.**
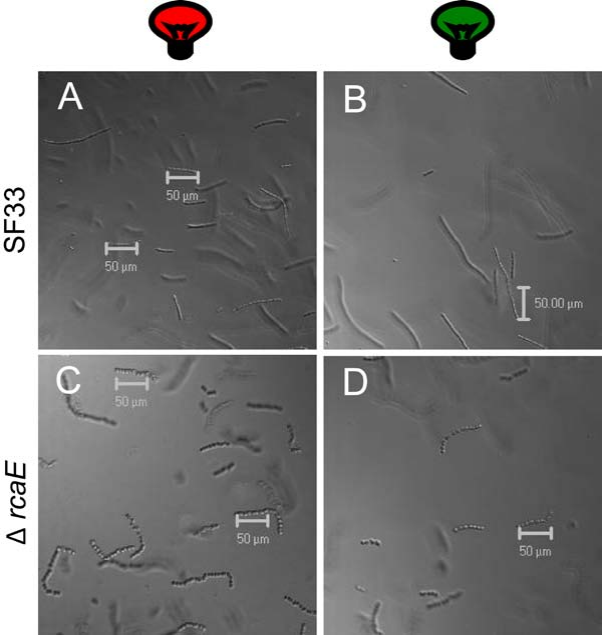
Differential Interference Contrast (DIC) images of filament populations of *Fremyella diplosiphon* strains grown in broad-band red and green light. Wild-type pigmentation SF33 (A, B) and Δ*rcaE* mutant (C, D) strains. Images at 20× dry objective. Bars, 50 μm.

CLSM is useful for detecting cellular and filament morphology in individual filaments as well as population scans (Figure 2) using DIC optics. To optimally capture PBP autofluorescence in *F. diplosiphon*, we performed spectral imaging using the Meta scan-head on the CLSM. We set image parameters using SF33 cells grown under either GL or RL conditions. A single filament was focused under a Plan-Apochromat 63× oil objective and utilized for image parameter determination. To detect PBP autofluorescence, the filament was excited with three individual laser wavelengths: 488 nm, 543 nm, or 633 nm. An emission spectrum was then gathered at 10.7-nm increments as a lambda Z-series.

**Figure 3.**
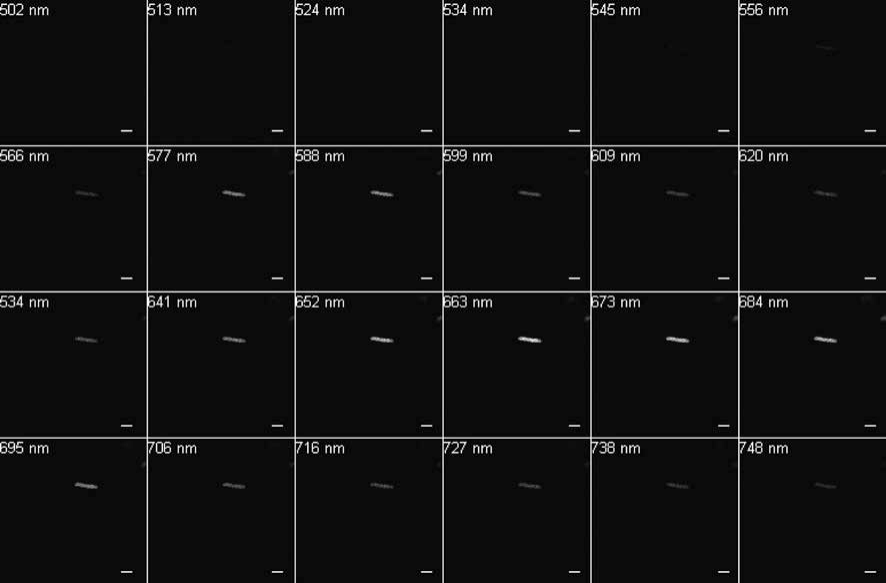
Spectral emission collection of SF33 GL-grown cells after excitation with 488-nm laser. An emission spectrum was gathered at 10.7-nm increments in the range from 500 nm to 750 nm. All images at 63× oil objective. Bars, 10 μm.

**Figure 4.**
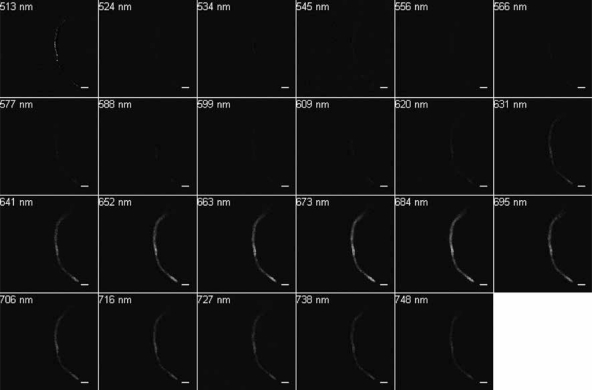
Spectral emission collection of SF33 RL-grown cells after excitation with 488-nm laser. An emission spectrum was gathered at 10.7-nm increments in the range from 500 nm to 750 nm. All images at 63× oil objective. Bars, 10 μm.

**Figure 5.**
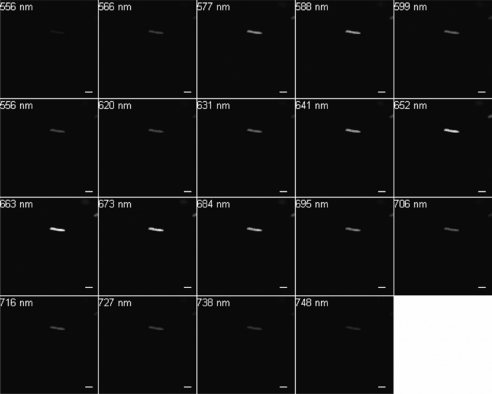
Spectral emission collection of SF33 GL-grown cells after excitation with 543-nm laser. An emission spectrum was gathered at 10.7-nm increments in the range from 550 nm to 750 nm. All images at 63× oil objective. Bars, 10 μm.

**Figure 6.**
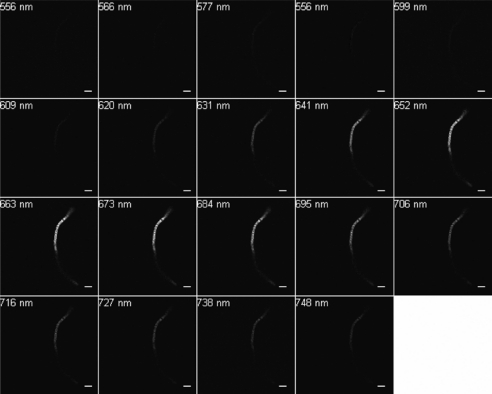
Spectral emission collection of SF33 RL-grown cells after excitation with 543-nm laser. An emission spectrum was gathered at 10.7-nm increments in the range from 550 nm to 750 nm. All images at 63× oil objective. Bars, 10 μm.

**Figure 7.**
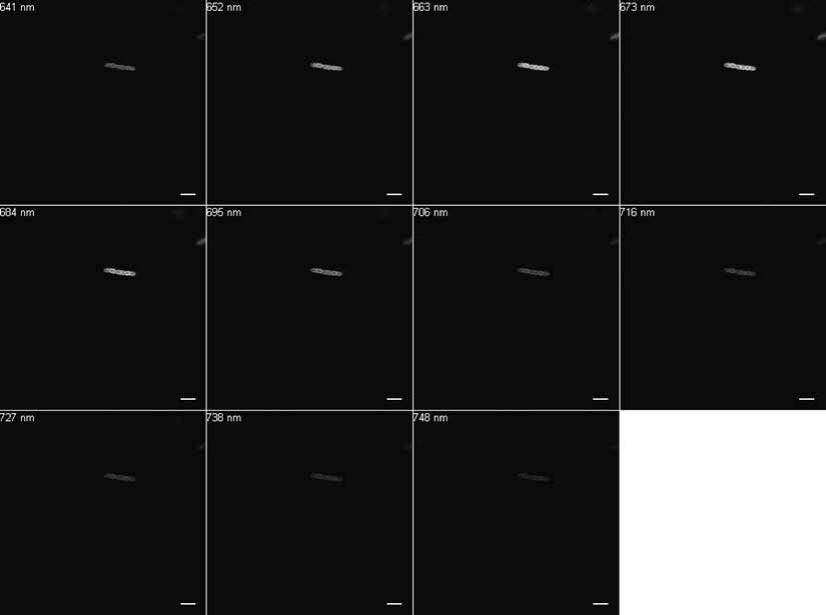
Spectral emission collection of SF33 GL-grown cells after excitation with 633-nm laser. An emission spectrum was gathered at 10.7-nm increments in the range from 640 nm to 750 nm all images at 63× oil objective. Bars, 10 μm.

**Figure 8.**
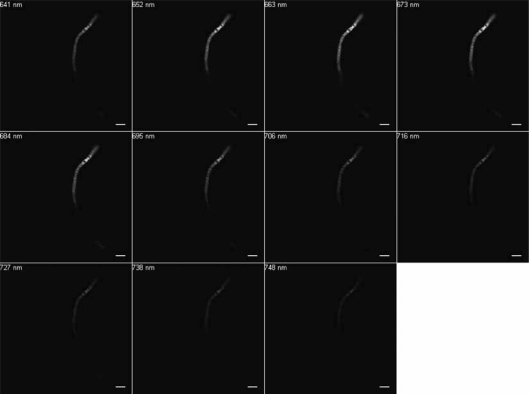
Spectral emission collection of SF33 RL-grown cells after excitation with 633-nm laser. An emission spectrum was gathered at 10.7-nm increments in the range from 640 nm to 750 nm. All images at 63× oil objective. Bars, 10 μm.

Excitation at 488 nm and scanning from 500 to 750 nm resulted in wavelengths of maximum emission at ∼580 and 650 nm for SF33 cells grown under GL (Figure 3) and at ∼670 nm for cells under RL (Figure 4). Excitation at 543 nm was associated with maximal emission at ∼580 and 660 nm for SF33 cells grown under GL (Figure 5) and at ∼660 nm for cells under RL (Figure 6) when emission scans were obtained in the range of ∼550 to 750 nm. Finally, excitation with the 633-nm laser and the obtaining of emission scans in the ∼640 to 750 nm range resulted in maximal emission at ∼660–670 nm under both GL (Figure 7) and RL (Figure 8).

The collected spectra were analyzed for wavelengths where fluorescence reached maximum emission (λmax; Table 1), as well as maximum relative fluorescence intensity. The ranges of maximum PBP autofluorescence and fluorescence intensity were compared between the three different excitation wavelengths in order to determine the best filter settings to use for subsequent imaging. Notably, λmax for emission was identical under GL and RL for excitation at 633. However, the collection of scans for this excitation necessitate the lowest wavelength as ∼640, which is above the maximal detection for PE found in cells under GL growth. Thus, this condition was considered suboptimal for detection of autofluorescence for GL-grown cells, and additionally the intensity for RL-grown cells was less than that observed for excitation at 488 or 543 nm. Comparing excitation at 488 and 543 nm, overall fluorescence was higher with 543 nm excitation under both GL and RL (Figures 5 and 6) as compared to excitation at 488 (Figures 3 and 4).

Based on these observations, final autofluorescence was collected using a 543-nm laser for excitation with a primary dichroic mirror (HFT 488/543), a secondary dichroic mirror (NFT 490) and finally a long pass (LP) 560-nm emission filter independent of light growth conditions of *F. diplosiphon* cells. Emission was collected using a 560- to 615-nm band pass (BP) filter, selected to capture fluorescence correlating with the accumulation of GL-inducible PE, and a 640- to 753-nm Meta detector, selected to capture fluorescence correlating with the accumulation of PCc, AP, and Chl *a* for GL-grown cells. Emission for RL-grown cells was collected with a 615-nm LP filter selected to capture fluorescence correlating with the accumulation of RL-inducible PCi, as well as PCc, AP, and Chl *a*.

### 3.2. Imaging different *F. diplosiphon* strains with optimal settings for detecting PBP autofluorescence

**Figure 9.**
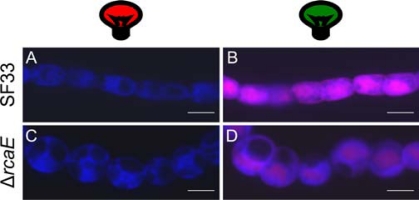
Phycobiliprotein autofluorescence of *Fremyella diplosiphon* strains grown in broad-band red and green light. Wild-type pigmentation SF33 (A, B) and Δ*rcaE* mutant (C, D) strains. Maximum-projection images from a Z-series of GL- and RL-adapted filaments were acquired at 63× oil objective with a 2× zoom setting. False colors are scaled equally among images. Bars, 5 μm.

These optimized CLSM settings were used to image SF33 and Δ*rcaE* strains grown under GL or RL to demonstrate the efficacy of detecting PBP autofluorescence and observing cellular and filament morphologies. In imaged samples, the fluorescence channels were falsely colored to reflect the PBS composition under each light condition; for RL-grown filaments, the autofluorescence observed using a 615-nm long pass filter is indicated by blue color, which correlates with the accumulation of PCi, as well as PCc, AP, and Chl *a* (Figure 9A and 9C). For GL-grown filaments, the autofluorescence observed using a band pass filter of 560–615 nm is indicated by pink, correlating with the accumulation of GL-inducible PE, as well as autofluorescence from PCc, AP, and Chl *a* using the meta scanning filter from 640–753 nm, indicated by blue color (Figure 9B and 9D). The distinctions in PBP composition are apparent in comparisons of SF33 cells grown in RL *vs.* GL (compare Figure 9A and 9B). The use of autofluorescence has also enhanced our ability to observe clear distinctions in cellular morphology between cells of SF33 and Δ*rcaE* strains (Figure 9; [2,19]).

## 4. Conclusions

The autofluorescent nature of proteins such as cyanobacterial PBPs and Chl *a* makes them useful as *in vivo* markers. These *in vivo* markers facilitate the use of non-invasive techniques including CLSM for studies with live cells. The distinct autofluorescent properties of PBPs makes them a good target for probing the regulation of their synthesis and accumulation [12], as well as effective markers for investigating the regulation of cellular shape and filament morphology *in vivo* in the organisms in which they are found [2,11,20], apart from their recognized utility as fluorescent tags in heterologous systems [9]. The CLSM technology has also been used for detecting PBP autofluorescence to associate distinct spectral characteristics with distinct developmental stages in cyanobacterial systems [7]. We have demonstrated that CLSM imaging can be optimized for detecting strain-specific proteins that accumulate differentially under distinct growth conditions to study the accumulation of the proteins or markers themselves, to illuminate cellular and/or organismal structures (e.g., cell shape or filament morphology), or to probe the role of particular genes and gene products in the regulation of defined cellular and/or organismal structures (e.g., the role of RcaE in the regulation of cell shape and filament morphology). Furthermore, the use of spectral scanning CLSM for mutants, in particular, may make possible the identification of mutants lacking particular autofluorescent proteins and allow for studies on the related genes and their roles in the regulation of pigmentation.

## Figures and Tables

**Table 1.** Wavelength(s) of maximum absorption after excitation with 488, 543, and 633 nm laser lines. Maximum ranges of fluorescence emission collected at 10.7-nm increments after excitation of GL- and RL-grown filaments.

Excitation laser (nm)	Scan range (nm)	λmax RL (nm)	λmax GL (nm)
488	∼500–750	∼670	∼580 & 650
543	∼550–750	∼660	∼580 & 660
633	∼640–750	∼660–670	∼660–670
